# The Interaction Between *Echinococcus multilocularis* Calreticulin S-Domain and Human Complement C1q Inhibits C1q-Dependent Immune Functions

**DOI:** 10.3390/pathogens15040427

**Published:** 2026-04-16

**Authors:** Meng Xia, Yinghui Song, Xiaofang Dong, Li Gu, Yishuo Wang, Wen Sun, Bin Zhan, Yan Yan, Limei Zhao

**Affiliations:** 1 Department of Pathogenic Biology, School of Basic Medical Sciences and Forensic Medicine, Baotou Medical College, Baotou 014040, China; 18574858035@163.com (M.X.); 13677153913@163.com (Y.S.); dxfsybb@163.com (X.D.); gulikangjing@163.com (L.G.); 13191528167@163.com (Y.W.); 15045320183@163.com (W.S.); 2 Department of Pediatrics, National School of Tropical Medicine, Baylor College of Medicine, Houston, TX 77030, USA; bzhan@bcm.edu

**Keywords:** *Echinococcus multilocularis*, calreticulin, binding site, S-domain, macrophages, immune evasion

## Abstract

*Em*CRT is a calreticulin secreted by *Echinococcus multilocularis* during its infection in host, playing an important role in evading host immune attack as a survival strategy. Our previous study has demonstrated that recombinant *Em*CRT (r*Em*CRT) was able to bind to C1q and lectin to interfere with host classical and lectin complement activation pathway, respectively. However, the C1q-binding site on *Em*CRT and the associated immune evasion mechanism remain unknown. In this study, the C1q-binding site on *Em*CRT was determined through molecular docking analysis and fragment expression to be localized to the S-domain (*Em*CRT-S) between Lys^140^ at the N-domain and Gln^292^ at the P-domain. The recombinant *Em*CRT-S protein (r*Em*CRT-S) was subsequently expressed in bacteria. Functional analysis confirmed that r*Em*CRT-S was able to bind to human C1q and inhibit C1q-initiated complement activation at the similar level to the full-length r*Em*CRT, resulting in the reduction in C4b/C3b deposition and antibody-sensitized sheep red blood cell hemolysis. Furthermore, r*Em*CRT-S binding to C1q suppressed THP-1-derived macrophage chemotaxis and ROS generation. Given that the identified functional domain *Em*CRT-S provides similar complement regulatory functions to the full-length *Em*CRT, this domain is a more feasible and practical target for vaccine development against *E. multilocularis* infection or for inflammatory and autoimmune diseases.

## 1. Introduction

Echinococcosis is a serious parasitic disease caused by the infection with larvae of tapeworm *Echinococcus*. It includes cystic echinococcosis caused by *Echinococcus granulosus* and alveolar echinococcosis caused by *E. multilocularis* [1,2]. Carnivores such as foxes, dogs and wolves act as definitive hosts for these parasites, harboring the mature tapeworm in their intestine. Rodents or sheep act as the intermediate hosts infected with larvae in their bodies. Humans become infected by accidentally ingesting the eggs released by the definitive hosts in their feces [3,4]. Infection of *E. multilocularis* larvae caused alveolar echinococcosis (AE) in liver that exhibits diffuse infiltrative growth within the hepatic parenchyma resembling malignant tumors. This leads the AE to a “hepatocellular carcinoma-like” fatal parasitic disease. AE is primarily prevalent in high-latitude countries of the Northern Hemisphere. Globally, it is estimated that more than 10 thousand new infections occur annually worldwide. China has one of the highest incidence rates globally, with most of the cases found in the northwest and southwest regions. Owing to the lack of validated therapeutic and preventive strategies, AE has not been well-controlled to date [5,6,7,8]. To survive in the host, the larvae of *E. multilocularis* (metacestode) have developed sophisticated tactics to evade host immune responses. Elucidating the immune evasion mechanism of the parasite within host will facilitate AE prevention and control [4]. Several studies revealed that *Echinococcus* parasites were able to secrete some proteins to target the host complement system as a survival strategy. For example, *E. granulosus* expressed an InsP6 protein component that binds to the host complement H factor [9] and *E. multilocularis* secreted proteins that act as complement inhibitors [10]. These results indicate *Echinococcus* parasites develop certain mechanism to interfere with host complement activation to escape the innate immunity attack.

The complement system is a key component of innate immunity, acting as a primary immune defense against invaded pathogens. An activated complement can form a membrane attack complex (MAC) on cell membranes, thereby killing invaded pathogens including parasites. The complement system activation proceeds through three pathways, namely the classical, lectin, and alternative pathways [11,12]. The classical pathway serves as an important effector mechanism in immune responses. C1q, the initial complement component of the classical pathway, consists of an N-terminal collagen-like domain and a C-terminal globular head domain (gC1q). It possesses both complement-dependent and complement-independent functions, including the regulation of the functions of multiple immune cells, such as macrophages, neutrophils, eosinophils and mast cells to facilitate their migration to infection or inflammation sites [13,14,15]. The complement-binding on macrophages also regulates their production of reactive oxygen species, thereby augmenting pathogen-clearing potential.

Calreticulin (CRT) is a calcium-binding chaperone protein serving an essential role in diverse biological processes. It contains a globular N-terminus, a proline-rich P-domain in the middle, and an acidic C-terminus. The C-domain contains negatively charged amino acids with Ca^2+^ binding activity [16,17]. Some helminth CRTs are able to bind to C1q thereby suppressing C1q-dependent complement activity. For example, nematode *Trichinella spiralis* produced calreticulin (*Ts*CRT) to interact with C1q and suppress the activation of classical complement pathway [18]. Furthermore, the C1q-binding region on *Ts*CRT had been localized to the S-domain, which encompasses amino acids within N- and P-domains [19]. Recent crystal structure analysis of *Ts*CRT has further elucidated its interaction with C1q at the atomic level, which revealed that C1q-binding region on *Ts*CRT shares the same region to bind on IgG. Based on the key residues involved in C1q-binding, a functionally engineered peptide P*^Ts^*^CRT^ was designed that exhibits potent C1q-binding activity and complements inhibitory capacity [20]. Similarly, calreticulin derived from the nematode *Brugia malayi (Bm*CRT) showed binding activity to C1q through its N- and P-domains to impede the C1q-mediated classical complement pathway [21], and *Haemonchus contortus* calreticulin (*Hc*CRT) employed its N-domain to interact with C1q to inhibit host complement activity as an immune escaping strategy [22]. All these results suggest that helminth-derived CRTs are involved in modulating host immune response, especially at innate complement activation level during parasite infection. Our previous study has successfully cloned calreticulin from *E. multilocularis* metacestode larvae (*Em*CRT), where immunization of recombinant *Em*CRT protein provided protective immunity against *E. multilocularis* infection in a mouse model [23]. Further studies demonstrated that *Em*CRT played an important role in interfering with multiple complement activation pathways through binding to C1q and lectin [24,25]. However, the specific C1q-binding site on *Em*CRT is still unclear. In this study, we predicted the specific C1q binding site on *Em*CRT based on molecular docking and interaction. The specific C1q-binding domain was determined as the S-domain of *Em*CRT (*Em*CRT-S). The recombinant *Em*CRT-S protein (r*Em*CRT-S) was expressed that exerted full immunoregulatory activities to suppress classical complement activation pathway and C1q-mediated monocyte-macrophage functions as the full-length *Em*CRT did. Identification of the C1q-binding site on *Em*CRT provides a molecular structural and functional basis for targeting this functional domain (*Em*CRT-S) as a candidate molecule for the anti-echinococcosis vaccine and therapeutic drug development.

## 2. Materials and Methods

### 2.1. Experimental Animals

Female BALB/c mice aged 6–8 weeks were provided by SPF Biotechnology Co., Ltd. (Beijing, China), and housed under specific pathogen-free conditions with controlled temperature and humidity. All experimental protocols performed in this study were reviewed and approved by the IACUC of Baotou Medical College (approval number: 2023-29, 27 November 2023). The study complies with the NIH Guidelines for the Care and Use of Laboratory Animals.

### 2.2. Sera

Normal human sera (NHS) used in this study were collected from six healthy volunteers approved by the Institutional Review Board (IRB) of Baotou Medical College (approval number: 202510-8, 30 November 2025). Human C1q-deficient serum (C1qD) was purchased from Quidel (San Diego, CA, USA).

### 2.3. Molecular Docking

The three-dimensional structure of *Em*CRT was modeled using AlphaFold 3 (DeepMind, London, UK) [25]. Molecular docking between *Em*CRT and the human complement element C1q (PDB: 1PK6) was established using the protein-protein docking tool HDOCK v1.0 (Wuhan University of Science and Technology, Wuhan, China).

### 2.4. Recombinant Proteins Preparations

**Figure 1:**
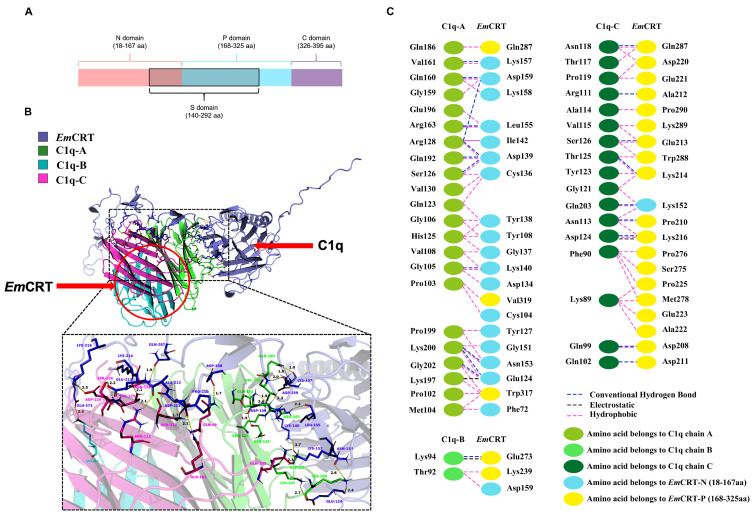
Molecular docking-predicted interaction of *Em*CRT and human C1q (PDB ID: 1PK6). (**A**) Schematic structure of *Em*CRT functional domains [25]. (**B**) Schematic diagram showing the predicted binding of the N- and P-domains of *Em*CRT to C1q. (**C**) Predicted interacting residues and bond types between *Em*CRT and C1q.

DNAs coding for full-length *Em*CRT (r*Em*CRT, 18–395 aa) and its N-domain (r*Em*CRT-N, 18–167 aa), P-domain (r*Em*CRT-P, 168–325 aa), NP-domain (r*Em*CRT-NP, 18–325 aa), C-domain (r*Em*CRT-C, 326–395 aa), and S-domain (r*Em*CRT-S, 140–292 aa) fragments (shown in Figure 1A) were amplified from *E. multilocularis* metacestode total cDNA and cloned into the prokaryotic expression vector pET-28a(+) (Novagen, Madison, WI, USA). The corresponding recombinant proteins were expressed in *E. coli* BL21 under induction of 1 mM IPTG. All recombinant proteins contain a 6-Histidin-tag at N-terminus and were purified by Ni^2+^ affinity chromatography (Beyotime Biotechnology, Shanghai, China) as described [25]. Endotoxin contamination in purified recombinant proteins was eliminated using the ToxinEraser™ Endotoxin Removal Kit (GenScript, Nanjing, China) and verified by the ToxinSensor Endotoxin Detection System (GenScript, Nanjing, China). The final protein concentration was quantified with the BCA Protein Assay Kit (Solarbio, Beijing, China).

### 2.5. Preparation of Polyclonal Antibody Against rEmCRT-S

Ten BALB/c mice were randomly divided into two groups with 5 each. Mice in the first group were subcutaneously immunized each with 25 μg of r*Em*CRT-S protein emulsified with Montanide™ ISA 50V2 adjuvant (Seppic, Paris, France) in a total volume of 100 µL (25 μg r*Em*CRT-S in 50 μL PBS emulsified with 50 μL adjuvant) three times with one week intervals. Another group of mice were injected with PBS + Montanide™ ISA 50V2 as a control. One week after the final immunization, all mice were euthanized and bloods were collected. The sera were isolated and IgG was purified using an rProtein G Beads 4FF affinity chromatography column (Solarbio, Beijing, China) to obtain polyclonal antibodies against r*Em*CRT-S. Antibody titers were determined by ELISA using r*Em*CRT-S-coated plates. The purified antibody was aliquoted and stored at −80 °C.

### 2.6. Identification of Human C1q-Binding Region in EmCRT

#### 2.6.1. ELISA

Different concentrations of human C1q complement (0, 0.05, 0.1, 0.2, 0.4, 0.8, 1.2, 1.5 μg/well) (Abcam, Cambridge, UK) in the coating buffer (100 mM Na_2_CO_3_/NaHCO_3_, pH 9.6) were coated on 96-well plates (100 μL/well) at 4 °C overnight. The same amount of bovine serum albumin (BSA) was coated on plates as a control. After being blocked with 3% BSA (200 μL), the plates were added with 100 µL of 50 nM r*Em*CRT, r*Em*CRT-NP, r*Em*CRT-S, r*Em*CRT-P, r*Em*CRT-C, and r*Em*CRT-N in the binding buffer (0.05% Triton-X 100, 20 mM Tris-HCl, 1 mM CaCl_2_, 50 mM NaCl, pH 7.4), respectively, and incubated overnight at 4 °C. Mouse anti-His monoclonal antibody (BOSTER, Chengmai, China) at 1:3000 dilutions and HRP-labeled goat anti-mouse IgG (BOSTER, Chengmai, China) at 1:10,000 dilutions were employed to detect the recombinant proteins bound on C1q coated on the plates. After incubation with chromogenic substrate TMB (Beyotime Biotechnology, Shanghai, China), the OD_450_ was read by an ELISA reader (Thermo Fisher Scientific, Waltham, MA, USA) to quantify the protein bound on the plate.

#### 2.6.2. Far Western Blot

A total of 5 µg of human C1q or BSA (5 μg) were run on each lane of 12.5% SDS-PAGE gels under denaturing conditions and then transferred onto PVDF membranes (Merck, Darmstadt, Germany). After being blocked with 5% skimmed milk in PBS, the membranes were incubated with r*Em*CRT, r*Em*CRT-NP, r*Em*CRT-N, r*Em*CRT-P, r*Em*CRT-C, or r*Em*CRT-S at the concentration of 5 μg/mL in the same binding buffer mentioned above at 4 °C overnight. The binding of recombinant proteins to C1q were determined using mouse anti-His monoclonal antibody (1:5000) and HRP-labeled goat anti-mouse IgG (1:10,000). The experiment was repeated independently three times.

#### 2.6.3. Immunoprecipitation

To further verify the binding ability of C1q to r*Em*CRT-S under non-denaturing conditions, 50 μL of Protein G MicroBeads (Miltenyi Biotec, Cologne, Germany) was incubated with 3 μg of anti-His mAb and 5 μg of r*Em*CRT-S on ice for 30 min in the presence or absence of 1 mM CaCl_2_. The incubation without r*Em*CRT-S was served as the negative control. After incubation, the beads were washed three times with washing buffer (50 mM Tris-HCl, 1% NP-40, 50 mM NaCl, pH 8.0) to remove unbound proteins. Afterwards the incubation mixture was mixed with 5 μg of human C1q and the incubation was continued at 4 °C overnight, then the beads were thoroughly washed with washing buffer six times. To elute the bound proteins on the beads, a total of 70 µL of preheated 1 × SDS sample buffer was added into each bead sample. The eluted proteins were separated on 12.5% SDS-PAGE gels and transferred onto PVDF membranes. The rabbit anti-human C1qA antibody (Abcam, Cambridge, UK) at dilutions of 1:5000 was used to determine the C1q bound to non-denatured r*Em*CRT-S.

### 2.7. rEmCRT-S Suppresses C1q-Triggered Classical Complement Activation Pathway

#### 2.7.1. Measurement of C4b and C3b Deposition

To determine the inhibition of r*Em*CRT-S on the C1q-initiated classical complement activation, 96-well plates were coated with human IgM (0.2 μg/well) as a C1q activation initiator at 4 °C overnight. The plates were washed with PBST (PBS + 0.05% Tween-20) and blocked with 5% BSA for 2 h at 37 °C. Next, 1 μg of C1q was pre-incubated with varying concentrations of r*Em*CRT-S (0.1 and 0.5 μM), r*Em*CRT (0.5 μM), or BSA (0.5 μM) in 100 µL for 2 h at 37 °C before being transferred to the IgM-coated plates for one more hour at 37 °C. After being washed with PBST, the plates were incubated with C1q-D at 1:200 dilutions in 1 × Veronal buffer (VB; Lonza, Switzerland) containing 0.05% Tween-20 and 0.1% gelatin at 37 °C for 1 h. NHS (1:50) was served as a positive control. After being washed with PBST three times, the levels of C4b and C3b deposited on the wells were determined by goat anti-human C4b polyclonal antibody (1:3000, Abcam, Cambridge, UK) and rabbit anti-human C3b monoclonal antibody (1:1000, BOSTER, Chengmai, China), respectively. HRP-conjugated secondary antibodies were used for detection, and the absorbance values were obtained at 450 nm using an ELISA reader.

#### 2.7.2. Hemolytic Assay

To determine whether r*Em*CRT-S affects classical complement activation-induced hemolysis, a total of 1 μg of C1q was incubated with different amounts of r*Em*CRT-S (1, 2, and 3 μM), r*Em*CRT (3 μM), or BSA (3 μM) in 100 μL binding buffer for 2 h at 37 °C. Then, 100 µL of C1q-D (1:100) in Hank’s balanced salt solution (HBSS^++^, Solarbio, Beijing, China) was added as supplement of complement except for C1q. NHS and inactivated serum were served as controls. Fresh sheep red blood cells at a concentration of 5 × 10^8^ cells/mL in 1 × HBSS^++^ were sensitized with rabbit anti-erythrocyte antibody (1:500; Zhengzhou Baiji Biotechnology Co., Ltd., Zhengzhou, China) in HBSS^++^ containing 1 mM MgCl_2_ and 0.15 mM CaCl_2_ at 37 °C for 30 min. The sensitized SRBC were added into the pre-incubated complex above and incubated at 37 °C for 30 min. The lysed supernatant was obtained after being centrifuged at 1500 rpm for 10 min. The hemolysis level was determined by measuring the absorbance at 412 nm in the supernatants against the total hemolysis in water.

### 2.8. rEmCRT-S Attenuates C1q-Induced M2 Macrophage Function

THP-1, a human leukemia monocyte cell line, was purchased from Procell Life Science & Technology Co., Ltd. (Wuhan, China). The cells were first differentiated into M0 macrophages by incubation in the RPMI 1640 medium supplemented with 10% fetal bovine serum (FBS; Procell, Wuhan, China) and 100 nM PMA (MedChemExpress, Monmouth Junction, NJ, USA) at 37 °C, 5% CO_2_, for 24 h. To induce M2 macrophages, the cells were further incubated with 20 ng/mL human IL-4 and IL-13 (PeproTech, Rocky Hill, NJ, USA) for 48 h.

Following induction, the M2 macrophage cells were collected and washed with PBS. The Fc receptor on the cell was blocked by incubating with Fc receptor blocker (Invitrogen, Carlsbad, CA, USA) for 30 min at 4 °C. Subsequently, the cells were stained with phycoerythrin (PE)-conjugated anti-human CD206 antibody (BioLegend, San Diego, CA, USA) and analyzed by flow cytometry, and the mean fluorescence intensity (MFI) of CD206-PE was determined [26,27].

#### 2.8.1. Suppression of C1q Binding to M2 Macrophages

To determine whether r*Em*CRT-S inhibits the binding of C1q to M2 macrophages, THP-1-derived M2 macrophages were allowed to adhere on coverslips in a 24-well plate (2 × 10^5^ cells/well). Following fixation with 4% paraformaldehyde at room temperature for 20 min, the cells on the coverslips were rinsed with PBS and blocked with normal goat serum for 30 min at room temperature. C1q (0.1 μM) was pre-incubated with various concentrations of endotoxin-free r*Em*CRT-S (5 and 10 μM), r*Em*CRT (10 μM), or the complex of r*Em*CRT-S (10 μM) with mouse anti-r*Em*CRT-S polyclonal antibody at 37 °C for 2 h before being transferred to the coverslips. After being washed with PBST, the cells on the coverslips were incubated with rabbit anti-C1q monoclonal antibody (1:100; Abcam, Cambridge, UK) at 4 °C overnight. The DyLight 488-labeled goat anti-rabbit IgG (1:200; BOSTER, Chengmai, China) was served as the secondary antibody. The cell nuclei were stained with DAPI. The stained cell images were acquired under a confocal laser scanning microscope (Nikon, Tokyo, Japan).

#### 2.8.2. Transwell Chemotaxis Assay

To investigate the impact of r*Em*CRT-S on C1q-mediated chemotactic migration of macrophages, Transwell assays were performed using 8 μm-pore inserts (Corning, NY, USA). THP-1-derived M2 were seeded (2 × 10^5^/well) into the upper chamber. Human C1q (10 nM) mixed with different concentrations of r*Em*CRT-S (1 and 2 μM), r*Em*CRT (2 μM), or a preformed complex of r*Em*CRT-S (2 μM) and mouse anti-r*Em*CRT-S polyclonal antibody were added to the lower chamber. LPS (100 ng/mL) and BSA (2 μM) were used as a control. After incubation at 37 °C under 5% CO_2_ for 24 h, non-migrated cells on the upper membrane surface were gently removed. The migrated cells on the bottom surface of the membranes were fixed with methanol and stained with 0.1% crystal violet. Cell numbers were counted in five randomly selected fields under microscopy.

#### 2.8.3. Detection of ROS Release

ROS levels were measured to examine whether r*Em*CRT-S inhibits C1q-induced M2 macrophage activity. C1q was coated onto 96-well plates (1 μg/well), then incubated with different concentrations of r*Em*CRT-S (0.5 and 1 μM), r*Em*CRT (1 μM), or the complex of r*Em*CRT-S (1 μM) with mouse anti-r*Em*CRT-S polyclonal antibody at 37 °C for 2 h. After washing with PBS, a total of 5 × 10^4^ M2 macrophages were added to each well and incubation continued for another 24 h. After washing, 100 μL of serum-free medium containing 10 μM DCFH-DA (Beyotime, Shanghai, China) was added to each well, followed by incubation at 37 °C in the dark for 30 min. The probe solution was then removed, and each well was washed three times with 200 μL PBS. The ROS level was measured using DCFH-DA with a fluorescence microplate reader set at 488 nm excitation and 525 nm emission.

#### 2.8.4. Statistical Analysis

All results were presented as means ± standard deviation. One-way or two-way ANOVA was used to analyze the statistical differences between different groups using GraphPad Prism 10 (San Diego, CA, USA). *p* < 0.05 was regarded as statistically significant. MFI values were calculated using FlowJo software (v10.8.1, BD Biosciences, San Jose, CA, USA) by Welch’s *t*-test.

## 3. Results

### 3.1. Prediction of C1q-Binding Region in EmCRT

Molecular docking between *Em*CRT and C1q was conducted using the predicted structure of *Em*CRT and the crystal structure of C1q (PDB ID: 1pk6) via the HDOCK server. The three-dimensional structure of *Em*CRT (GenBank: CUT99434.1) was predicted using AlphaFold3. VERIFY3D assessment revealed that 87.85% of residues had a 3D-1D score ≥ 0.1, confirming acceptable structural quality. Molecular docking was performed using HDOCK, generating 100 conformations; the conformation with a docking score of −461.58 kcal/mol was selected for subsequent analysis. This conformation exhibits a favorable binding mode, and the docking score suggests effective binding between the two molecules. The results showed that amino acid residues mainly located within the N- and P-domains of *Em*CRT participated in binding to C1q (Figure 1A,B): specifically, the amino acids Phe^72^, Cys^104^, Tyr^108^, Glu^124^, Tyr^127^, Asp^134^, Cys^136^, Gly^137^, Tyr^138^, Asp^139^, Lys^140^, Ile^142^, Gly^151^, Asn^153^, Leu^155^, Lys^157^, Lys^158^, Asp^159^ in the N-domain, and Gln^287^, Trp^317^, Val^319^ in the P-domain interacted with Pro^A102^, Pro^A103^, Met^A104^, Gly^A105^, Gly^A106^, Val^A108^, Gln^A123^, His^A125^, Ser^A126^, Arg^A128^, Val^A130^, Gly^A159^, Gln^A160^, Val^A161^, Arg^A163^, Gln^A186^, Gln^A192^, Glu^A196^, Lys^A197^, Pro^A199^, Lys^A200^, and Gly^A202^ of the C1q A chain. The amino acids Asp^159^ in the N-domain, and Lys^239^ and Glu^273^ in the P-domain interacted with Thr^B92^ and Lys^B94^ of the C1q B chain. The amino acid Lys^152^ in the N-domain and Asp^208^, Pro^210^, Asp^211^, Ala^212^, Glu^213^, Lys^214^, Lys^216^, Asp^220^, Glu^221^, Ala^222^, Glu^223^, Pro^225^, Ser^275^, Pro^276^, Met^278^, Gln^287^, Trp^288^, Lys^289^, and Pro^290^ in the P-domain interacted with Lys^C89^, Phe^C90^, Gln^C99^, Gln^C102^, Arg^C111^, Asn^C113^, Ala^C114^, Val^C115^, Thr^C117^, Asn^C118^, Pro^C119^, Gly^C121^, Tyr^C123^, Asp^C124^, Thr^C125^, Ser^C126^ and Gln^C203^ of the C1q C chain (Figure 1C). Interaction analysis indicated that the binding of *Em*CRT to C1q was mainly mediated by hydrogen bonds and electrostatic and hydrophobic interactions. Docking predictions suggest that the C1q-binding region in *Em*CRT is primarily located between Phe^72^ in the N-domain and Val^319^ in the P-domain.

### 3.2. Localization of the Human C1q-Binding Region in EmCRT

**Figure 2:**
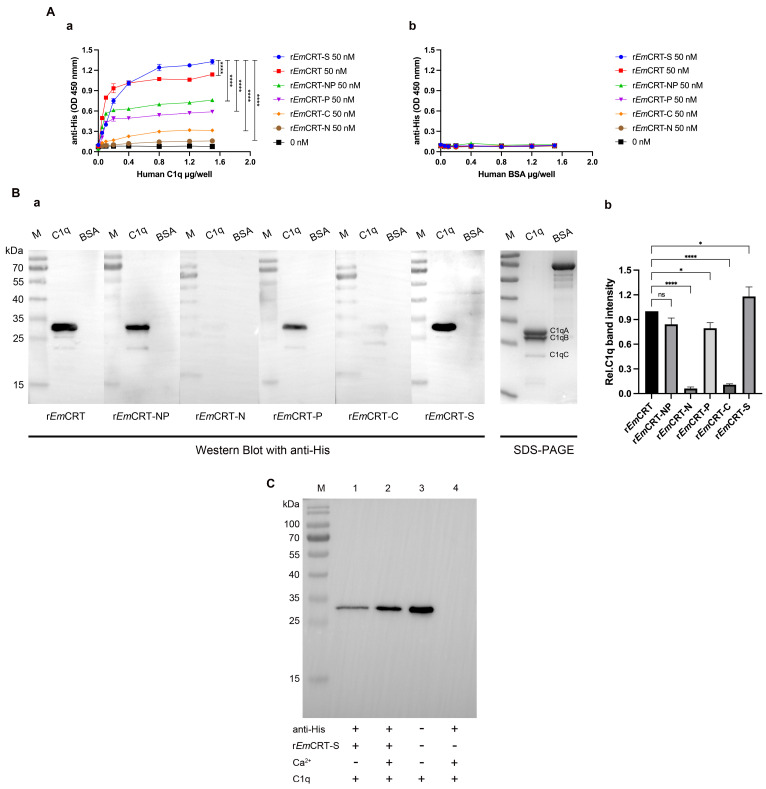
Binding capacity of different r*Em*CRT fragments to human C1q. (**A**) ELISA analysis of the binding between different r*Em*CRT fragments and human C1q. Bound fragments were detected using an anti-His antibody (**a**). No significant binding was observed in the BSA-coated control wells (**b**). Each experiment was repeated three times. Data are presented as mean ± SDs. Statistical comparisons were performed using two-way ANOVA with Tukey’s post hoc test. Asterisks indicate comparisons among fragments at the C1q concentration of 1.5 μg/well (**** *p* < 0.0001). (**B**) Far Western blot analysis of C1q binding to r*Em*CRT and its fragments. C1q and BSA (each 5 μg) were transferred onto PVDF membranes, then incubated with 5 μg/mL of r*Em*CRT or its fragments and probed with an anti-His antibody. The right panel shows SDS-PAGE of the same amount of C1q and BSA (**a**). Densitometric analysis of the detected bands is shown in (**b**), with the value for r*Em*CRT set to 1.0. Data are presented as mean ± SDs. Statistical comparisons were performed using one-way ANOVA with Tukey’s post hoc test (ns, no significant difference; * *p* < 0.05, **** *p* < 0.0001). (**C**) Immunoprecipitation assay for Ca^2+^-dependent binding of r*Em*CRT-S to C1q. C1q was pulled down by immunoprecipitation with r*Em*CRT-S immobilized on anti-His IgG/Protein G beads in buffers with or without Ca^2+^ (1 mM). The pull-down protein complex was separated by SDS-PAGE, transferred to a PVDF membrane, and detected using a rabbit anti-human C1qA antibody. Lane 3, loaded with 5 μg of human C1q as positive control. M, molecular weight marker.

The C1q-binding region in *Em*CRT was determined by different assays. ELISA results showed that r*Em*CRT, as well as r*Em*CRT-S, r*Em*CRT-NP and r*Em*CRT-P, was able to bind to C1q in a C1q dose-dependent manner, reaching saturating binding when C1q concentration was 0.8 μg/well. Notably, at the C1q concentration of 1.5 μg/well, r*Em*CRT-S exhibited significantly higher binding affinity to C1q compared to r*Em*CRT, r*Em*CRT-NP and r*Em*CRT-P. However, r*Em*CRT-N and r*Em*CRT-C revealed relatively low affinity to C1q even when the concentration of C1q reached to 1.5 µg/well. These results suggest the C1q-binding site of *Em*CRT is located within *Em*CRT-S (Figure 2(Aa)). In contrast, BSA showed no obvious binding to any of the r*Em*CRT fragments under the same condition even when the concentration of BSA reached to 1.5 µg/well (Figure 2(Ab)).

To further compare the relative binding strength of these fragments to C1q, Far Western blot analysis was performed. Following SDS-PAGE and the transfer of C1q onto PVDF membranes, the membranes were incubated with r*Em*CRT or its fragments. Bound fragments were detected using anti-His antibody. Consistent with the ELISA results, the binding capacity of r*Em*CRT-S to C1q is similar to or even stronger than r*Em*CRT, and significantly higher than r*Em*CRT-P and r*Em*CRT-NP (Figure 2(Ba)), which was further confirmed by densitometric analysis, with the value for r*Em*CRT set to 1.0 (Figure 2(Bb)). There was no significant binding of r*Em*CRT-N and r*Em*CRT-C to C1q observed. Both ELISA and Far Western blotting results verify that the C1q-binding region of *Em*CRT is located in *Em*CRT-S within the r*Em*CRT-NP fragment.

To determine whether Ca^2+^ is essential for r*Em*CRT-S binding to C1q, immunoprecipitation was conducted at the non-denatured condition. After co-incubating r*Em*CRT-S with C1q in buffers with or without Ca^2+^, the protein complex was pulled down by anti-His antibody immobilized on Protein G MicroBeads. Western blot analysis with anti-C1q antibody on the pull-down complex demonstrated that the binding ability between r*Em*CRT-S and C1q (most C1qA chain) was markedly increased by adding Ca^2+^. In the control group, the anti-His antibody alone (without r*Em*CRT-S) failed to pull down C1q, excluding the possibility of non-specific binding (Figure 2C).

### 3.3. rEmCRT-S Suppresses C1q-Triggered Classical Complement Activation

**Figure 3:**
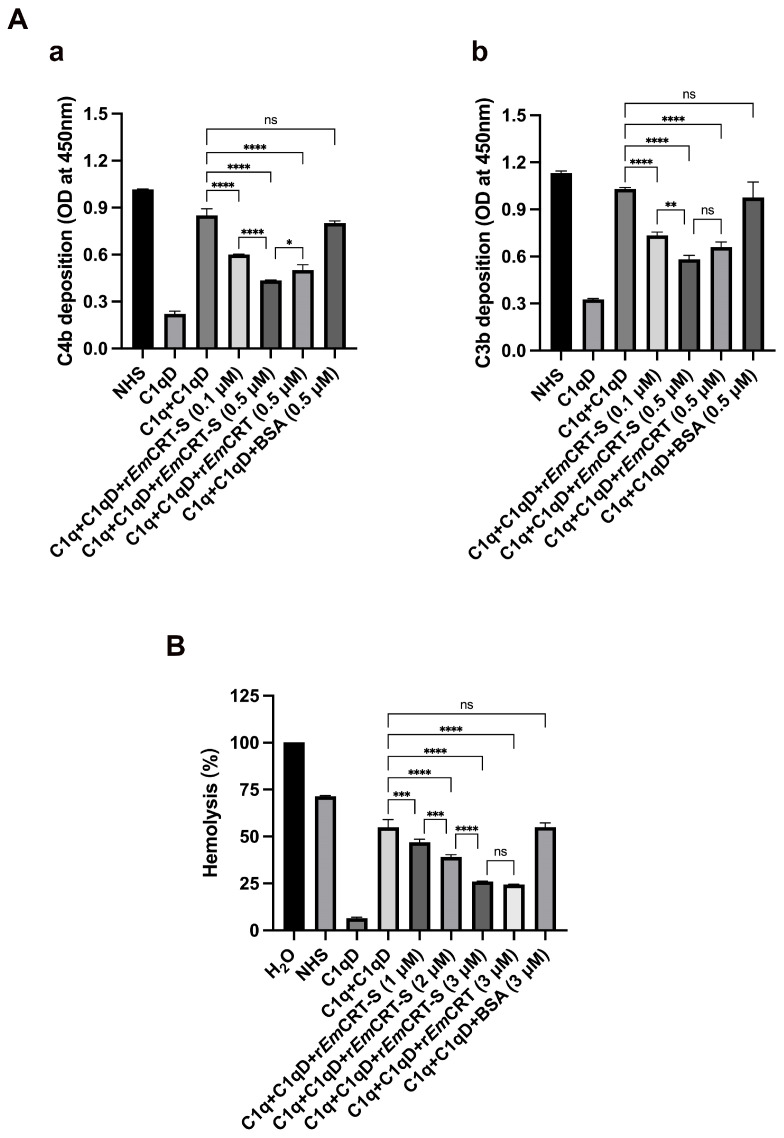
r*Em*CRT-S inhibits classical complement activation. (**A**) r*Em*CRT-S inhibits C4b and C3b deposition. C1q was pre-incubated with r*Em*CRT-S (0.1 and 0.5 μM), r*Em*CRT (0.5 μM), or BSA (0.5 μM) and then added to human IgM-coated wells. After C1qD addition, C4b (**a**) and C3b (**b**) deposition were detected using anti-C4b and anti-C3b antibodies, respectively. (**B**) r*Em*CRT-S inhibits complement-mediated hemolysis. C1q was pre-incubated with r*Em*CRT-S (1, 2, and 3 μM), r*Em*CRT (3 μM), or BSA (3 μM) before adding into antibody-sensitized sheep blood cells and C1qD. Erythrocyte lysis rate was determined by measuring the absorbance of the supernatant. Data are presented as mean ± SDs from three independent experiments. Statistical comparisons were performed using one-way ANOVA with Tukey’s post hoc test (ns, no significant difference; * *p* < 0.05; ** *p* < 0.01; *** *p* < 0.001; **** *p* < 0.0001).

To assess whether r*Em*CRT-S inhibits classical complement activation through binding to C1q, ELISA was used to detect the deposition of C4b and C3b, the intermediate product of classical complement activation. The results showed the classical complement activation was successfully initiated by IgM-C1q and reconstituted by adding C1qD serum (BSA was added as non-relevant protein control), reaching the level comparable to NHS based on the detection of C3b and C4b deposited on the plates. However, co-incubation of C1q with increasing doses of r*Em*CRT-S significantly reduced C4b (Figure 3(Aa)) and C3b (Figure 3(Ab)) deposition in a dose-dependent manner. In three independent experiments at a concentration of 0.5 μM, r*Em*CRT-S inhibited C4b and C3b deposition by 48.8% and 43.5%, respectively, whereas full-length r*Em*CRT showed inhibition rates of 41.0% and 36.1%. In contrast, BSA showed no inhibitory activity. As expected, the C1qD alone (without supplemental C1q) showed a significantly low level of C4b/C3b deposition. These results demonstrated that r*Em*CRT-S inhibited classical complement pathway activation by binding to C1q at the similar to or even better than full-length r*Em*CRT. To further evaluate the functional consequence of this inhibition, a complement-mediated hemolysis assay was performed. As shown in Figure 3B, r*Em*CRT-S as well as r*Em*CRT significantly inhibited antibody-initiated sheep erythrocyte lysis in a dose-dependent manner. r*Em*CRT-S and full-length r*Em*CRT at 3 μM inhibited complement-mediated hemolysis by 52.5% and 55.5% with no statistical significance. In contrast, the same amount of BSA exhibited no inhibitory activity. C1qD alone failed to induce significant hemolysis in the absence of C1q. These results confirm that r*Em*CRT-S specifically inhibits complement-mediated hemolysis by binding to C1q.

### 3.4. rEmCRT-S Inhibits C1q Binding to Macrophage

**Figure 4:**
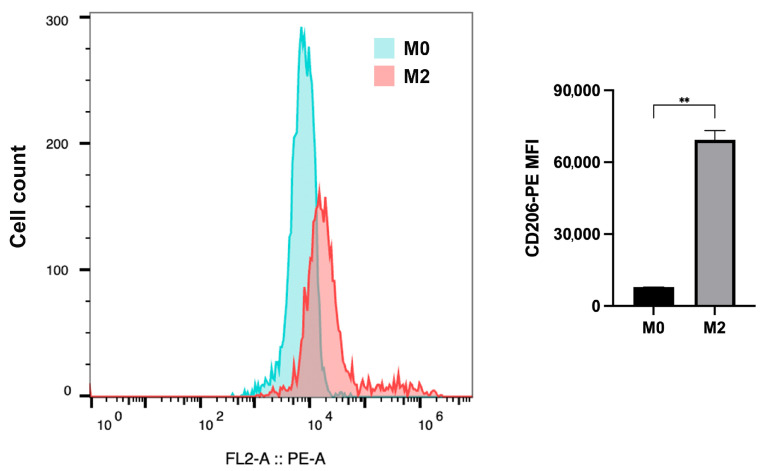
Flow cytometric analysis of CD206 expression in M0 and M2 macrophages. The left panel shows representative histograms of CD206-PE fluorescence intensity, with blue and pink curves representing M0 and M2 macrophages, respectively. The right panel presents quantitative analysis of the MFI of CD206 from three independent experiments. MFI values were calculated using FlowJo and compared by Welch’s *t*-test. Data are presented as mean ± SDs (** *p* < 0.01).

**Figure 5:**
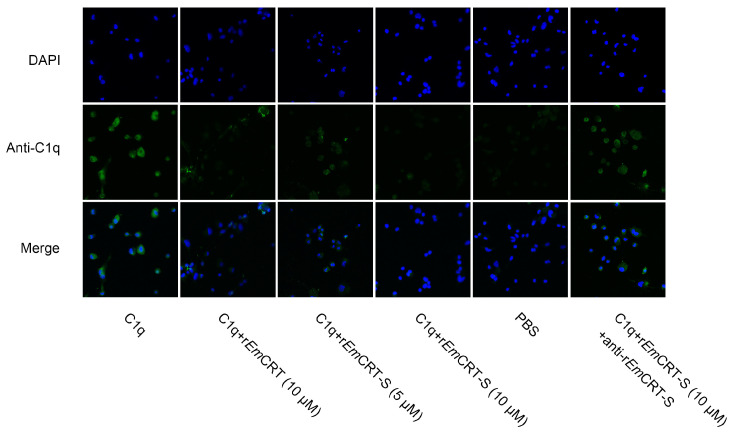
r*Em*CRT-S inhibits C1q binding to THP-1-derived M2 macrophages. M2 macrophages were treated with C1q (0.1 μM) pre-incubated with r*Em*CRT-S (5 and 10 μM), r*Em*CRT (10 μM), or r*Em*CRT-S (10 μM) and incubated with anti-r*Em*CRT-S antibody. C1q binding onto M2 cells was then detected using an anti-C1q primary antibody and a Dylight 488-labeled secondary antibody (green); nuclei were counterstained with DAPI (blue). Magnification: 400×.

M2 macrophages were differentiated from THP-1 cells induced by PMA, IL-4, and IL-13. The differentiation efficiency was confirmed by measuring the mean fluorescence intensity (MFI) of CD206 on the macrophage surface (Figure 4). To investigate the effect of r*Em*CRT-S on the binding of C1q to macrophage surface receptors, immunofluorescence staining with anti-C1q antibody was performed to detect C1q binding to cells. The results showed distinct green fluorescence around cells in the C1q-only group, whereas the C1q on the surface of M2 macrophages was significantly decreased in the group of co-incubation with r*Em*CRT-S in a dose-dependent manner. No obvious fluorescence was observed in the PBS negative control group. Notably, the addition of anti-r*Em*CRT-S antibody to the C1q-r*Em*CRT-S co-incubation complex significantly restored anti-C1q fluorescence intensity (Figure 5). These results indicate that r*Em*CRT-S inhibits the binding of C1q to the macrophage surface through its interaction with C1q. Neutralizing r*Em*CRT-S with a specific antibody demolishes its inhibition on C1q binding on M2 macrophages.

### 3.5. rEmCRT-S Inhibits C1q-Induced Macrophage Functions

**Figure 6:**
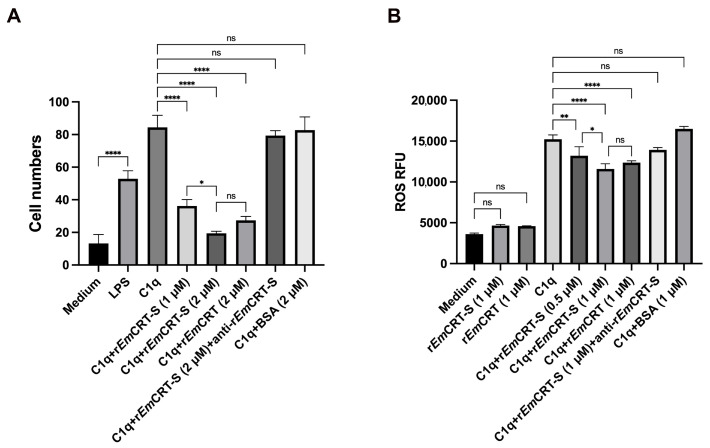
r*Em*CRT-S inhibited C1q-induced M2 macrophage migration and functions. (**A**) r*Em*CRT-S inhibited C1q-induced macrophage migration. C1q was pre-incubated with different concentrations of r*Em*CRT-S (1 and 2 μM) before adding to the lower chamber of a Transwell insert, and the number of cells that migrated to the underside of the membrane was counted. LPS (100 ng/mL) and C1q (1 μg/well) alone were added as controls. (**B**) r*Em*CRT-S inhibits C1q-induced macrophage secretion of ROS. C1q was pre-incubated with different concentrations of r*Em*CRT-S (0.5 and 1 μM) before adding to the plates. ROS fluorescence intensity was detected using the DCFH-DA probe. Each experiment was repeated three times. Data are presented as mean ± SDs. Statistical comparisons were performed using one-way ANOVA with Tukey’s post hoc test (ns, no significant difference; * *p* < 0.05; ** *p* < 0.01; **** *p* < 0.0001).

We further examined whether r*Em*CRT-S affected macrophage function by inhibiting C1q binding. Transwell assays were performed to evaluate the C1q-induced chemotactic migration capacity of M2 macrophages. The results showed that C1q significantly induced the migration of M2 macrophages. The addition of r*Em*CRT-S significantly reduced C1q-induced macrophage migration to the lower chamber in a dose-dependent manner (Figure 6A). At a concentration of 2 μM, r*Em*CRT-S inhibited C1q-induced macrophage migration by 76.9% compared to the r*Em*CRT at 2 μM that inhibited it by 67.5%. Furthermore, the addition of anti-r*Em*CRT-S antibody to the C1q/r*Em*CRT-S mixture restored macrophage cell migration to the level comparable to C1q alone. The group of BSA, the irrelevant protein control, had no significant effect on cell migration. As expected, LPS as a positive control promoted significant migration of macrophages, whereas the medium control (negative control) showed no chemotactic effect. In summary, these findings demonstrate that r*Em*CRT-S binds to C1q resulting in significantly reduced C1q-induced macrophage migration.

We next assessed the impact of r*Em*CRT-S binding to C1q on C1q-induced macrophage secretion of reactive oxygen species (ROS). C1q was pre-incubated with different concentrations of r*Em*CRT-S prior to the addition to THP-1 cells, and ROS generation was measured using the DCFH-DA fluorescent probe. The results showed that C1q alone significantly stimulated ROS production in macrophages, while ROS level was decreased when r*Em*CRT-S was added into C1q in a dose-dependent manner (Figure 6B). At a concentration of 1 μM, r*Em*CRT-S decreased ROS levels by 23.8%, that is higher than that induced by the full-length r*Em*CRT 19.0%, however the difference was not statistically significant. Moreover, the addition of the anti-r*Em*CRT-S antibody reversed the inhibitory effect of r*Em*CRT-S on the production of ROS to that comparable with C1q treatment alone. No significant inhibition was observed in the BSA control group. These findings indicate that r*Em*CRT-S interferes with C1q’s binding to macrophages, thereby inhibiting C1q-induced ROS release by macrophages.

## 4. Discussion

Over the course of long-term evolution, parasites have developed complex and sophisticated immune evasion mechanisms, among which the secretion of immunomodulatory molecules to disrupt host defense systems represents a key survival strategy [28,29]. As a vital component of the innate immune system, the complement system plays a pivotal role in combating the infections of invaded pathogens including parasites. Activated complement components not only directly lyse pathogens by forming membrane attack complexes but also enhance the clearance capacity of various immune cells (such as macrophages, neutrophils, mast cells, and eosinophils) through their opsonization effects [30]. C1q, as the initiator of the classical complement pathway, forms the C1 complex with C1r and C1s, and its functional activity is essential for triggering the complement activation cascade. CRT is a highly conserved calcium-binding protein that maintains calcium homeostasis and assists in protein folding [31]. Extracellularly, it participates in complement inhibition, enhancement of phagocytosis, and immune regulation. CRT from different species can either suppress or activate immune responses, aiding parasites in evading immunity while also serving as a vaccine candidate [17]. Recent studies have shown that CRTs derived from various parasites can bind to C1q, inhibiting its role in mediating complement activation and regulating immune cells, thereby aiding parasites in evading host immune attacks [18,19]. Furthermore, evidence indicates that calreticulin of helminth *Schistosoma japonicum* (SjCRT) could induce the maturation of dendritic cells and facilitate a Th1-skewed immune response in mice [32].

In our previous study, the *Em*CRT was cloned from *E. multilocularis* and its location was identified on the cyst wall of the metacestode, the surface of protoscoleces and in excretory-secretory products of *E. multilocularis* larvae; immunization with recombinant *Em*CRT (r*Em*CRT) provided significant protection against *E. multilocularis* infection in mice [23]. The expressed r*Em*CRT was further determined to exhibit high-affinity binding to C1q and inhibit both classical and lectin complement activation pathways and immune cell functions [24]. Additionally, we successfully predicted the three-dimensional structure of *Em*CRT [25], providing a structural basis for subsequent functional investigations. On this basis, the present study aims to localize the C1q-binding domain on *Em*CRT to facilitate the development of vaccine and therapy against the fatal infection in humans and livestock.

Molecular docking results predict that the region responsible for C1q binding in *Em*CRT is primarily located between Phe^72^ in the N-domain and Val^319^ in the P-domain. *Em*CRT interacts with C1q mainly through multiple amino acid residues within its N-terminal and P-domains via various interaction forces, including hydrogen bonds, electrostatic interactions, and hydrophobic interactions (Figure 1), which is similar to the fragment of *Ts*CRT-S [20]. Subsequent experiments confirmed that both r*Em*CRT and r*Em*CRT-S were able to bind to C1q at the similar level, that is significantly higher than other fragments (Figure 2). These results are consistent with previous studies reporting that the C1q-binding site of calreticulin is primarily located in the S-domain [19,33], confirming that r*Em*CRT-S contains the key domain for C1q binding. Immunoprecipitation experiments demonstrated that Ca^2+^ enhanced r*Em*CRT-S binding to C1q, consistent with the results of the crystallographic study that revealed the Ca^2+^ ion was necessary in maintaining the conformation of C1q globular head required for C1q binding to the IgG-Fc segment [34]. Furthermore, as a classic Ca^2+^-binding protein, CRT relies on Ca^2+^ binding to stabilize its tertiary structure and facilitate its interactions with corresponding ligands [35,36]. The strong binding capacity to C1q may confer r*Em*CRT-S as an intermediator to interfere with C1q-initiated complement activation and C1q-related immune cell functions.

At the functional level, in this study we identified that r*Em*CRT-S significantly inhibited C1q-mediated classical complement activation with significantly lower C4b and C3b deposition on a plate coated with IgM and C1q in a dose-dependent manner, with inhibitory effects comparable to full-length r*Em*CRT. Antibody-triggered sheep erythrocyte hemolysis assays provided additional validation of r*Em*CRT-S’s inhibitory effect on C1q-initiated complement activation (Figure 3). In addition, in our previous study, r*Em*CRT-S has also been shown to bind mannose-binding lectin (MBL), thereby inhibiting activation of the lectin complement pathway [25]. These observations suggest that *Em*CRT-S is the functional domain for interference with multiple complement pathways simultaneously, at least including classical and lectin complement activation pathways. However, the binding of r*Em*CRT-S to C1q could not completely inhibit C1q-initiated complement activation, possibly because the binding of r*Em*CRT-S could not completely abrogate C1 complex assembly.

Macrophages contain complement receptor 1 (CR1) and other receptors on their surface to interact with complement components [14]. Previous studies have shown that C1q is able to bind directly to CR1 and induce macrophage activation [37]. In the present study, we verified the binding of C1q to the surface of THP-1-derived M2 macrophages, the major phenotype of macrophage involved in the immune regulation and tissue repair. Treatment with r*Em*CRT-S markedly decreased C1q binding to macrophages (Figure 5), indicating that r*Em*CRT-S may exert its inhibitory activity on C1q/CR1-related macrophage functions. As we know, C1q binding to CR1 promotes macrophage migration to infectious or inflammatory sites with functions of tissue repair and pathogen clearance [38]. Indeed our study demonstrated that r*Em*CRT-S significantly inhibited C1q-induced macrophage migration in a dose-dependent manner. The addition of anti-r*Em*CRT-S dramatically reduced r*Em*CRT-S’s inhibitory ability to C1q-induced macrophage chemotaxis. These results indicate that C1q acts as a chemokine to recruit macrophages to inflammatory sites, while parasite-derived *Em*CRT (through *Em*CRT-S) plays a role in immune evasion by blocking C1q not only to inhibit host C1q-initiated complement activation (classical and lectin pathways) but also to interfere with the recruitment of immune cells to infection sites. Reactive oxygen species (ROS) production is a crucial mechanism for macrophage-mediated pathogen elimination, in addition to its role in chemotaxis to macrophage and other immune cells [39]. This study revealed that r*Em*CRT-S also inhibited C1q-induced ROS production of macrophages which was reversed by adding anti-r*Em*CRT-S antibody (Figure 6). These results indicate that r*Em*CRT-S binds to C1q to inhibit macrophages to produce ROS, thereby reducing their pathogen killing capacity and creating favorable conditions for parasite survival within the host. However, we did not measure whether reduced ROS was directly related to the reduced macrophage migration in this study.

In this study, the THP-1 cell line was used as an in vitro model to measure the C1q-binding induced macrophage-like effect; however, its differentiation and functional responses may differ from those of primary macrophages in vivo, and it may not fully recapitulate the dynamic changes during parasitic infection. Moreover, in vitro-induced M2 macrophages lack interactions with other immune cells, which may limit a full assessment of C1q-mediated functions. Therefore, the conclusions of this study require further validation in primary macrophages in vivo.

In summary, our results identified that the C1q-binding region of *Em*CRT is located in *Em*CRT-S between Lys^140^ at the N-domain and Gln^292^ at the P-domain, which exhibits an equivalent C1q-binding ability to the full-length *Em*CRT. r*Em*CRT-S not only inhibits multiple C1q-initiated complement activation pathways (classical and lectin) but also influences macrophage migration and oxidative bursts by blocking C1q-macrophage interactions. Since the full-length *Em*CRT has been demonstrated to exert partial protective effects against *E. multilocularis* infection with a 43.16% reduction in larval number [23]. The identification of a functional domain on *Em*CRT facilitates and accelerates the development of a preventive vaccine or therapeutic drug against *E. multilocularis* infection. It is also beneficial for developing a drug for complement-associated autoimmune disorders or other inflammatory diseases. The proof of concept of this functional domain design has been successfully tested using the functionally engineered peptide P*^Ts^*^CRT^ that provided the comparable inhibitions on C1q-related complement activation to its parental from *Ts*CRT [20]. However, there is a concern for developing *Em*CRT or its C1q-binding domain as vaccine or anti-inflammatory drug targets due to its sequence similarity with host CRT (50% amino acid sequence identity with human calreticulin) that may cause cross-reactivity and autoimmune injury. In fact, *Trypanosoma cruzi* CRT induced cross-reactive antibodies targeting host CRT and caused subsequent tissue injury [40,41]. Although cross-reactivity of anti-*Em*CRT antibodies with human CRT and related pathological changes during *E. multilocularis* infection have not been verified, this potential risk requires strict assessment for the safe development of *Em*CRT-based vaccines or drugs. Further investigations should be performed to assess the cross-reactivity of anti-*Em*CRT or C1q-binding domain with human CRT and human tissue to ensure its safety for vaccine or drug development. We expect that the functional epitope, such as *Em*CRT-S identified in this study, may be beneficial for reducing the side effects induced by the whole molecule including a potential cross-reaction with host tissue [20].

## Data Availability

Data included in this article are available from the corresponding author upon request.

## References

[1] Taratuto A.L., Venturiello S.M. (1997). Echinococcosis. Brain Pathol..

[2] Yan W.L., Meng J.X., Li X.M., Zhao J.P., Zhang M., Wang X.Y., Sun Y.Z., Ni H.B., Ma H. (2022). Global Prevalence of Echinococcosis in Goats: A Systematic Review and Meta-Analysis. Foodborne Pathog. Dis..

[3] Eckert J., Deplazes P. (2004). Biological, epidemiological, and clinical aspects of echinococcosis, a zoonosis of increasing concern. Clin. Microbiol. Rev..

[4] McSorley H.J., Hewitson J.P., Maizels R.M. (2013). Immunomodulation by helminth parasites: Defining mechanisms and mediators. Int. J. Parasitol..

[5] Rostami A., Lundstrom-Stadelmann B., Frey C.F., Beldi G., Lachenmayer A., Chang B.C.H., Norouzian M.M., Hemphill A., Gasser R.B. (2025). Human Alveolar Echinococcosis-A Neglected Zoonotic Disease Requiring Urgent Attention. Int. J. Mol. Sci..

[6] Mitra S., Charaya P., Deshpande S.G., Parkhi M., Yadav T.D. (2024). Hepatic alveolar echinococcosis simulating metastatic malignancy. Autops. Case Rep..

[7] Wen H., Vuitton L., Tuxun T., Li J., Vuitton D.A., Zhang W., McManus D.P. (2019). Echinococcosis: Advances in the 21st Century. Clin. Microbiol. Rev..

[8] Paternoster G., Boo G., Wang C., Minbaeva G., Usubalieva J., Raimkulov K.M., Zhoroev A., Abdykerimov K.K., Kronenberg P.A., Mullhaupt B. (2020). Epidemic cystic and alveolar echinococcosis in Kyrgyzstan: An analysis of national surveillance data. Lancet Glob. Health.

[9] Irigoin F., Laich A., Ferreira A.M., Fernandez C., Sim R.B., Diaz A. (2008). Resistance of the *Echinococcus granulosus* cyst wall to complement activation: Analysis of the role of InsP6 deposits. Parasite Immunol..

[10] Qiu Y., Shen S., Yang Y., Wang W. (2022). An Excretory Protein of *Echinococcus multilocularis* Inhibits Complement Classical Pathway Activation. Infect. Drug Resist..

[11] Dunkelberger J.R., Song W.C. (2010). Complement and its role in innate and adaptive immune responses. Cell Res..

[12] Heggi M.T., Nour El-Din H.T., Morsy D.I., Abdelaziz N.I., Attia A.S. (2023). Microbial evasion of the complement system: A continuous and evolving story. Front. Immunol..

[13] Reid K.B.M. (2018). Complement Component C1q: Historical Perspective of a Functionally Versatile, and Structurally Unusual, Serum Protein. Front. Immunol..

[14] Nayak A., Pednekar L., Reid K.B., Kishore U. (2012). Complement and non-complement activating functions of C1q: A prototypical innate immune molecule. Innate Immun..

[15] Benoit M.E., Clarke E.V., Morgado P., Fraser D.A., Tenner A.J. (2012). Complement protein C1q directs macrophage polarization and limits inflammasome activity during the uptake of apoptotic cells. J. Immunol..

[16] Michalak M., Groenendyk J., Szabo E., Gold L.I., Opas M. (2009). Calreticulin, a multi-process calcium-buffering chaperone of the endoplasmic reticulum. Biochem. J..

[17] Esperante D., Flisser A., Mendlovic F. (2023). The many faces of parasite calreticulin. Front. Immunol..

[18] Zhao L., Shao S., Chen Y., Sun X., Sun R., Huang J., Zhan B., Zhu X. (2017). *Trichinella spiralis* Calreticulin Binds Human Complement C1q As an Immune Evasion Strategy. Front. Immunol..

[19] Shao S., Hao C., Zhan B., Zhuang Q., Zhao L., Chen Y., Huang J., Zhu X. (2020). *Trichinella spiralis* Calreticulin S-Domain Binds to Human Complement C1q to Interfere With C1q-Mediated Immune Functions. Front. Immunol..

[20] Jia Z., Yu W., Li J., Zhang M., Zhan B., Yan L., Ming Z., Cheng Y., Tian X., Shao S. (2024). Crystal structure of *Trichinella spiralis* calreticulin and the structural basis of its complement evasion mechanism involving C1q. Front. Immunol..

[21] Yadav S., Gupta S., Selvaraj C., Doharey P.K., Verma A., Singh S.K., Saxena J.K. (2014). In silico and in vitro studies on the protein-protein interactions between *Brugia malayi* immunomodulatory protein calreticulin and human C1q. PLoS ONE.

[22] Suchitra S., Joshi P. (2005). Characterization of *Haemonchus contortus* calreticulin suggests its role in feeding and immune evasion by the parasite. Biochim. Biophys. Acta.

[23] Chen L., Cheng Z., Xian S., Zhan B., Xu Z., Yan Y., Chen J., Wang Y., Zhao L. (2022). Immunization with *Em*CRT-Induced Protective Immunity against *Echinococcus multilocularis* Infection in BALB/c Mice. Trop. Med. Infect. Dis..

[24] Xian S., Chen L., Yan Y., Chen J., Yu G., Shao Y., Zhan B., Wang Y., Zhao L. (2023). *Echinococcus multilocularis* Calreticulin Interferes with C1q-Mediated Complement Activation. Trop. Med. Infect. Dis..

[25] Shao Y., Xia M., Song Y., Yan Y., Dong X., Zong H., Zhan B., Wang Y., Zhao L. (2025). *Echinococcus multilocularis* Calreticulin Inhibits Lectin Pathway of Complement Activation by Directly Binding to Mannose-Binding Lectin. Pathogens.

[26] Siniyeh A.A., Alshaer W., Elzogheir N., Al-Holi M., Alqudah D.A., Abuarqoub D., Kwiatek J.M. (2025). Comparative analysis of RT-qPCR, flow cytometry, and Di-4-ANEPPDHQ fluorescence for distinguishing macrophages phenotypes. Biochem. Biophys. Rep..

[27] Genin M., Clement F., Fattaccioli A., Raes M., Michiels C. (2015). M1 and M2 macrophages derived from THP-1 cells differentially modulate the response of cancer cells to etoposide. BMC Cancer.

[28] Gazzinelli-Guimaraes P.H., Nutman T.B. (2018). Helminth parasites and immune regulation. F1000Research.

[29] Hewitson J.P., Grainger J.R., Maizels R.M. (2009). Helminth immunoregulation: The role of parasite secreted proteins in modulating host immunity. Mol. Biochem. Parasitol..

[30] Shao S., Sun X., Chen Y., Zhan B., Zhu X. (2019). Complement Evasion: An Effective Strategy That Parasites Utilize to Survive in the Host. Front. Microbiol..

[31] Michalak M., Corbett E.F., Mesaeli N., Nakamura K., Opas M. (1999). Calreticulin: One protein, one gene, many functions. Biochem. J..

[32] Ma L., Li D., Yuan C., Zhang X., Ta N., Zhao X., Li Y., Feng X. (2017). SjCRT, a recombinant *Schistosoma japonicum* calreticulin, induces maturation of dendritic cells and a Th1-polarized immune response in mice. Parasit. Vectors.

[33] Stuart G.R., Lynch N.J., Lu J., Geick A., Moffatt B.E., Sim R.B., Schwaeble W.J. (1996). Localisation of the C1q binding site within C1q receptor/calreticulin. FEBS Lett..

[34] Gaboriaud C., Juanhuix J., Gruez A., Lacroix M., Darnault C., Pignol D., Verger D., Fontecilla-Camps J.C., Arlaud G.J. (2003). The crystal structure of the globular head of complement protein C1q provides a basis for its versatile recognition properties. J. Biol. Chem..

[35] Wijeyesakere S.J., Gafni A.A., Raghavan M. (2011). Calreticulin is a thermostable protein with distinct structural responses to different divalent cation environments. J. Biol. Chem..

[36] Venkatesan A., Satin L.S., Raghavan M. (2021). Roles of Calreticulin in Protein Folding, Immunity, Calcium Signaling and Cell Transformation. Prog. Mol. Subcell. Biol..

[37] Bobak D.A., Frank M.M., Tenner A.J. (1988). C1q acts synergistically with phorbol dibutyrate to activate CR1-mediated phagocytosis by human mononuclear phagocytes. Eur. J. Immunol..

[38] Alvarez-Dominguez C., Carrasco-Marin E., Leyva-Cobian F. (1993). Role of complement component C1q in phagocytosis of *Listeria monocytogenes* by murine macrophage-like cell lines. Infect. Immun..

[39] Tavassolifar M.J., Vodjgani M., Salehi Z., Izad M. (2020). The Influence of Reactive Oxygen Species in the Immune System and Pathogenesis of Multiple Sclerosis. Autoimmune Dis..

[40] Ribeiro C.H., Lopez N.C., Ramirez G.A., Valck C.E., Molina M.C., Aguilar L., Rodriguez M., Maldonado I., Martinez R., Gonzalez C. (2009). *Trypanosoma cruzi* calreticulin: A possible role in Chagas’ disease autoimmunity. Mol. Immunol..

[41] Eggleton P., Llewellyn D.H. (1999). Pathophysiological roles of calreticulin in autoimmune disease. Scand. J. Immunol..

